# Light Intensity and Gibberellic Acid Differentially Shape Primary Metabolism and Ginsenoside Profiles in *Panax ginseng* Sprouts

**DOI:** 10.3390/ijms27219614

**Published:** 2026-10-27

**Authors:** Seonghye Kim, Bo-Ram Choi, Hyeon Seon Na, Jin-Kyu Jang, Seung-Yeob Song, Jung-Woo Lee, Young Seob Lee, Kwan-Woo Kim, Dae Young Lee, Dahye Yoon

**Affiliations:** 1 Department of Herbal Crop Research, National Institute of Horticultural and Herbal Science, Rural Development Administration, Eumseong 27709, Republic of Korea; ksh0110@korea.kr (S.K.); bmcbr@korea.kr (B.-R.C.); youngseoblee@korea.kr (Y.S.L.); swamp1@korea.kr (K.-W.K.); 2 BK21 FOUR KNU Creative BioResearch Group, School of Life Sciences, Kyungpook National University, Daegu 41566, Republic of Korea; hs1295@knu.ac.kr (H.S.N.); jkjang80@knu.ac.kr (J.-K.J.); dylee80@knu.ac.kr (D.Y.L.); 3 Department of Food Sciences, National Institute of Crop and Food Science, Rural Development Administration, Wanju 55365, Republic of Korea; s2y337@korea.kr; 4 Department of Horticultural Crop Research, National Institute of Horticultural and Herbal Science, Rural Development Administration, Muan 58545, Republic of Korea; enzymer@korea.kr

**Keywords:** *Panax ginseng*, ginseng sprouts, metabolomics, gibberellic acid, light intensity, ginsenosides, primary metabolism

## Abstract

Ginseng sprouts (*Panax ginseng* C.A. Meyer) are mostly cultivated under controlled indoor conditions, allowing modulation of growth and phytochemical composition through environmental and hormonal treatments. In this study, ^1^H NMR and UPLC-QTOF/MS were used to investigate the independent and interactive effects of light intensity and gibberellic acid (GA_3_) on primary metabolism and ginsenoside profiles. While GA_3_ promoted shoot growth, high light reduced plant height but increased root dry weight. Multivariate analysis of 42 identified primary metabolites and 26 ginsenosides revealed that the primary metabolite profiles are primarily distinguished by GA_3_, whereas ginsenoside profiles are more clearly differentiated based on light intensity. GA_3_ broadly increased soluble carbohydrates and decreased free amino acids, whereas light intensity had a selective effect on some primary metabolites. In contrast to the limited interaction effects in primary metabolism, significant interactions between light and GA_3_ were detected for eight ginsenosides. High-intensity light increased the content of several ginsenosides, including Re, Rg1, Rd, Rb2, and Rc, while decreasing protopanaxadiol (PPD)-type ginsenosides that lack a sophorosyl moiety at C-3. Correlation analysis across the experimental conditions revealed coordinated variation between primary metabolites and ginsenosides, with the most prominent pattern corresponding to the light-response axis. Overall, GA_3_ and light intensity exerted distinct but interconnected effects across metabolic layers, providing a framework for understanding how cultivation factors determine growth and phytochemical composition in ginseng sprouts.

## 1. Introduction

Korean ginseng (*Panax ginseng* C.A. Meyer) is one of the most widely consumed medicinal plants in East Asia, recognized for its diverse pharmacological properties including immunomodulatory, anti-diabetic, neuroprotective, and anti-fatigue effects [1,2,3]. These bioactive effects are primarily attributed to ginsenosides, a class of triterpenoid saponins unique to the genus *Panax*. Ginseng sprouts, which are young whole plants harvested after 4–6 weeks of indoor cultivation, have emerged as a commercially promising form of *P. ginseng* [4]. Because ginseng sprouts are consumed as whole plants, including their ginsenoside-rich leaves, they offer greater potential as a functional food ingredient than conventional ginseng products derived solely from roots [5]. Conventional ginseng cultivation not only requires a long cultivation period, typically 4–6 years, but is also constrained by limited suitable cultivation areas due to soil fatigue associated with continuous cropping [6]. In contrast, ginseng sprouts are primarily cultivated under controlled indoor conditions, allowing precise regulation of environmental factors such as temperature, humidity, light intensity, and nutrient supply [7].

Controlled indoor cultivation of ginseng sprouts provides opportunities to modify their phytochemical composition through targeted cultivation practices. Among environmental factors, light plays a critical role in regulating photosynthesis, plant growth, and secondary metabolite accumulation in *P. ginseng*. The light environment has been identified as a key factor influencing biomass accumulation and ginsenoside content in ginseng sprouts, with white LED lighting reported to enhance root productivity and ginsenoside accumulation depending on its spectral composition [8,9]. Previous transcriptomic and metabolomic analyses have suggested that high light intensity promotes ginsenoside biosynthesis in *P. ginseng* leaves, accompanied by the upregulation of genes involved in carbon fixation and the tricarboxylic acid (TCA) cycle [10]. In addition to environmental factors, plant growth regulators such as gibberellic acid (GA_3_) play important roles in regulating plant growth and development. GA_3_ is widely known to promote shoot elongation and vegetative growth [11] and has also been reported to enhance seed germination, shoot growth, and the accumulation of saponins and other bioactive compounds in American ginseng sprouts [12]. Light and gibberellin signaling are closely interconnected in plants [13], and their interaction can also influence secondary metabolism. For example, GA_3_ has been shown to completely inhibit red light-induced flavonol glycoside synthesis in pea [14]. In tobacco, both light intensity and GA_3_ increased lignin deposition, although some light-dependent responses could not be reproduced by GA_3_ treatment alone [15]. However, the combined effects of light intensity and GA_3_ treatment in *P. ginseng* remain poorly understood.

Primary metabolism provides essential carbon skeletons and energy for the biosynthesis of specialized metabolites, while environmental and developmental factors can influence both primary and specialized metabolic pathways [16]. In *Panax* species, metabolomic studies have revealed substantial variation in sugars, amino acids, and organic acids across developmental stages, tissues, and growing seasons, often accompanied by changes in ginsenoside accumulation [17,18]. More recently, Yun et al. [19] reported harvest-time- and tissue-dependent variation in primary metabolites and ginsenosides in wild-simulated *P. ginseng*, along with significant correlations between several sugar metabolites and individual ginsenosides. These findings highlight the importance of considering ginsenoside accumulation in relation to primary metabolic status rather than as an isolated phytochemical response. However, despite increasing evidence of associations between primary and secondary metabolism in *Panax* species, the combined effects of controllable cultivation factors on these metabolic processes in ginseng sprouts remain poorly understood. Therefore, in the present study, an integrated metabolomics approach combining ^1^H NMR spectroscopy and UPLC-QTOF/MS was employed to investigate ginseng sprouts cultivated under two light intensities with or without GA_3_ treatment. This study aimed to evaluate the main and interactive effects of light intensity and GA_3_ on primary metabolite and ginsenoside profiles and to explore the relationships between primary and specialized metabolism.

## 2. Results

### 2.1. Growth Responses to Light Intensity and GA_3_ Treatment

Ginseng sprouts showed distinct growth responses to light intensity and GA_3_ treatment (Table 1 and Figure S1). Two-way ANOVA revealed significant main effects of GA_3_ on plant height and shoot dry weight, both of which increased following GA_3_ treatment. Light intensity significantly affected plant height and root dry weight, with high light intensity associated with reduced plant height and increased root dry weight. Leaf number tended to increase following GA_3_ treatment, although this effect was not statistically significant after correction for multiple testing. No significant interactions between light intensity and GA_3_ were observed for any growth parameter.

### 2.2. Multivariate Analysis of Primary Metabolites and Ginsenosides

**Figure 1:**
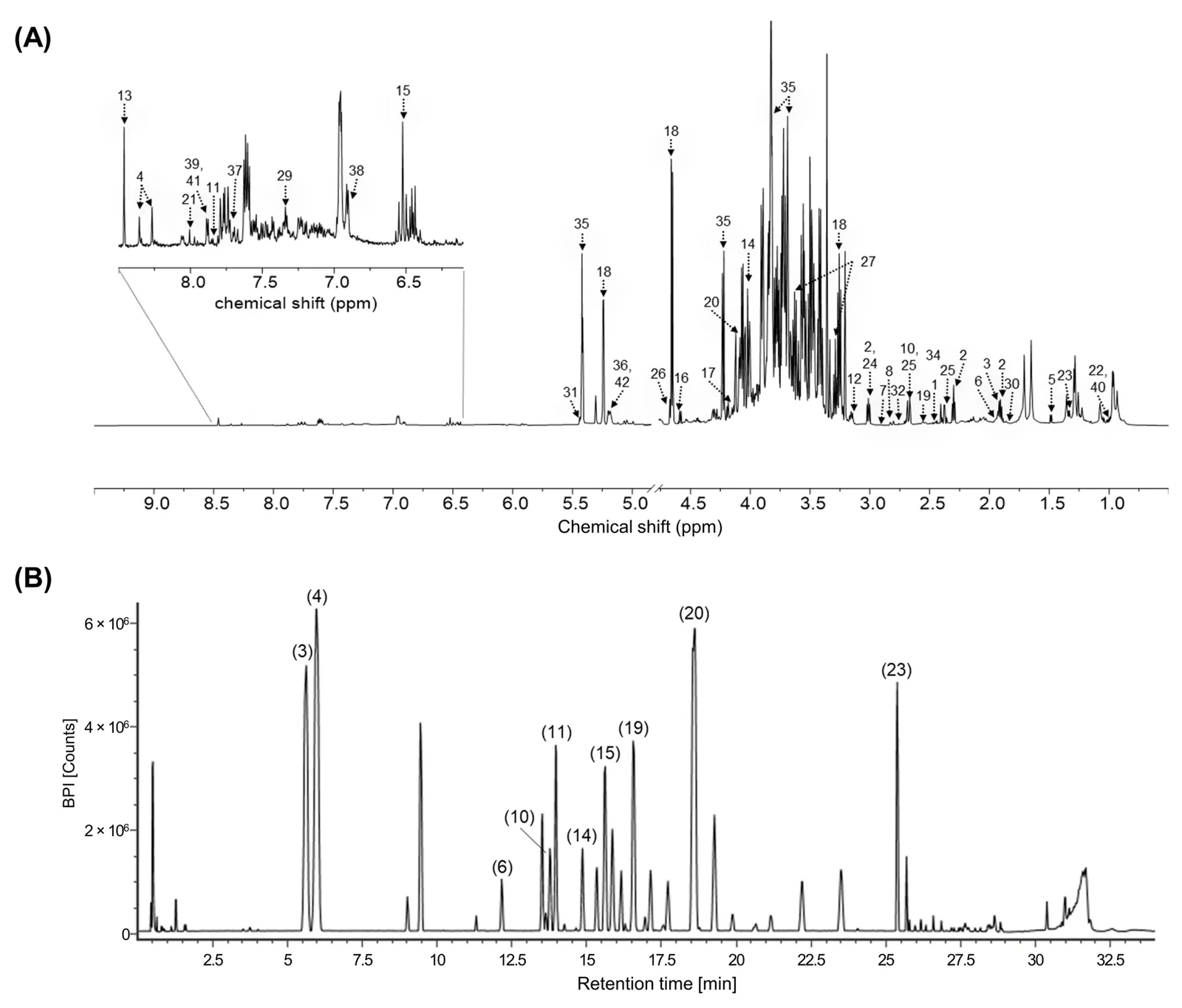
Representative ^1^H NMR spectrum (**A**) and UPLC-QTOF/MS base peak intensity (BPI) chromatogram (**B**) of ginseng sprout extracts. Compound numbers in (**A**) and (**B**) correspond to those listed in Table S1 and Table 2, respectively.

A total of 42 primary metabolites and 26 ginsenosides were identified by ^1^H NMR spectroscopy and UPLC-QTOF/MS, respectively (Table S1 and Table 2). The identified ginsenosides were classified into PPD-, protopanaxatriol (PPT)-, and oleanane-type ginsenosides. Representative ^1^H NMR spectra and base peak intensity (BPI) chromatograms are presented in Figure 1.

**Figure 2:**
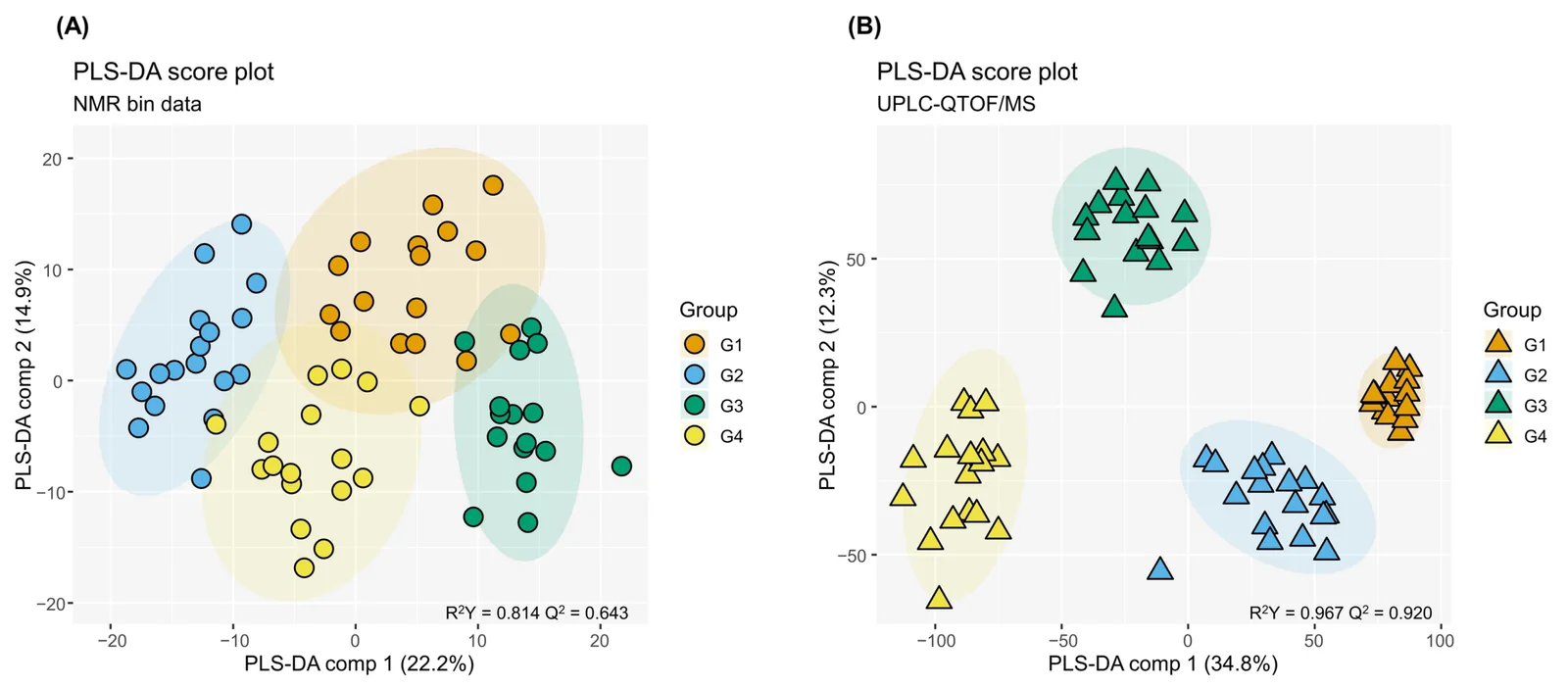
PLS-DA score plots of the ^1^H NMR (**A**) and UPLC-QTOF/MS (**B**) datasets. Shaded ellipses represent 95% confidence regions. R^2^Y and Q^2^ indicate model fit and predictive ability, respectively. G1, 200 μmol m^−2^ s^−1^ without GA_3_; G2, 200 μmol m^−2^ s^−1^ with GA_3_; G3, 400 μmol m^−2^ s^−1^ without GA_3_; G4, 400 μmol m^−2^ s^−1^ with GA_3_.

Partial least squares discriminant analysis (PLS-DA) was performed to assess differences in the overall metabolic profiles of ginseng sprouts grown under different light intensities and GA_3_ treatments (Figure 2). Cross-validation and permutation testing supported the predictive performance and statistical validity of both PLS-DA models, with Q^2^ values of 0.643 and 0.920 for the ^1^H NMR and UPLC-QTOF/MS datasets, respectively, and significant permutation test results (pR^2^Y and pQ^2^ = 0.01, 100 permutations, Figure S2). In the ^1^H NMR dataset, the first two components explained 37.1% of the total variation, with Component 1 and Component 2 accounting for 22.2% and 14.9%, respectively (Figure 2A). The four groups exhibited partially overlapping but distinguishable distributions. In particular, GA_3_-treated groups (G2 and G4) tended to cluster toward the negative side of Component 1, whereas the corresponding untreated groups (G1 and G3) were positioned toward the positive side, suggesting that GA_3_ treatment was associated with differences in primary metabolite profiles.

The UPLC-QTOF/MS dataset exhibited clearer separation among the four experimental groups than the ^1^H NMR dataset (Figure 2B). Components 1 and 2 explained 34.8% and 12.3% of the variation in the metabolite data, respectively. The four groups formed distinct clusters, with separation primarily associated with light intensity along Component 1. Overall, the PLS-DA results suggested that primary metabolite profiles were predominantly separated by GA_3_ treatment, whereas ginsenoside profiles were mainly separated by light intensity.

### 2.3. Effects of GA_3_ and Light Intensity on Primary Metabolites

**Figure 3:**
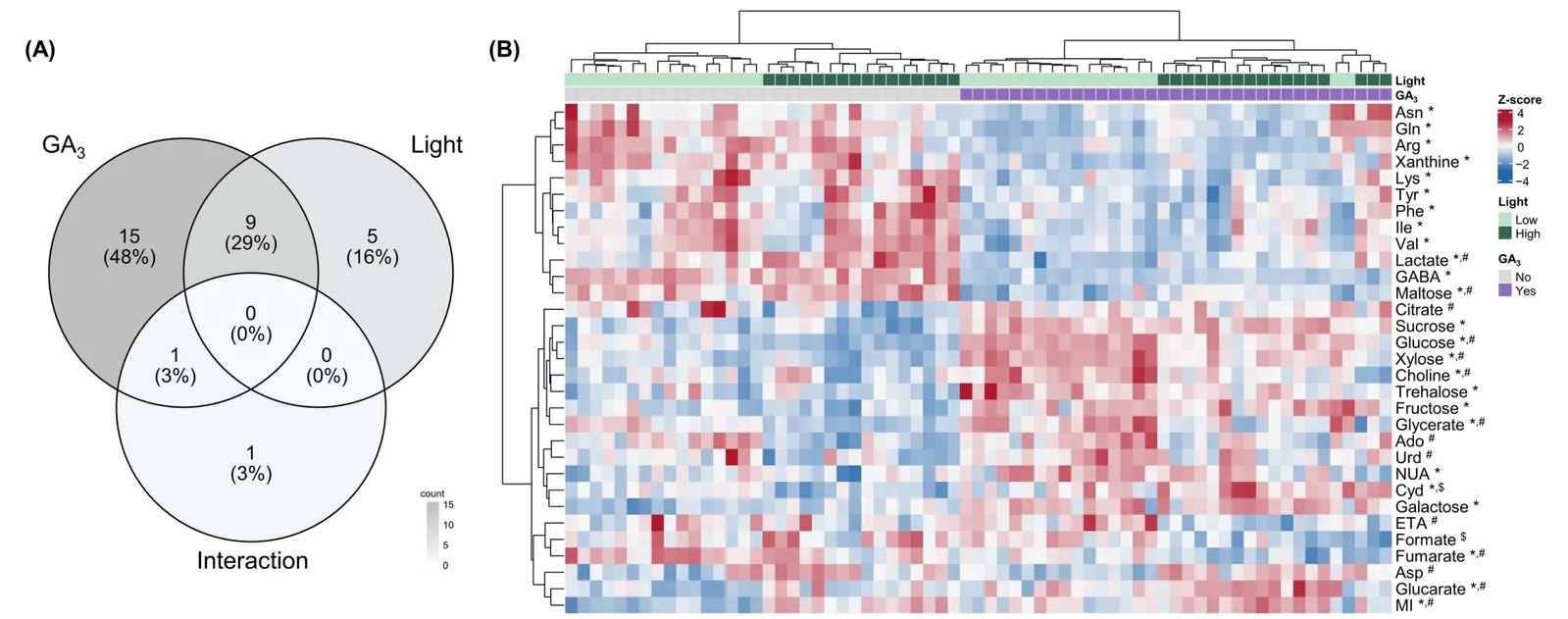
Two-way ANOVA results and heatmap of significantly affected primary metabolites identified by ^1^H NMR. (**A**) Venn diagram showing the numbers of metabolites significantly affected by GA_3_ treatment, light intensity, and their interaction. (**B**) Heatmap of significantly affected metabolites. Metabolite levels were scaled by row (Z-score). *, significant GA_3_ effect; #, significant light effect; $, significant interaction (adjusted *p* < 0.05). Metabolite abbreviations are defined in Table S1.

The effects of GA_3_ treatment and light intensity on individual primary metabolites were evaluated using two-way ANOVA (Figure 3A and Table S1).

Of the 42 identified primary metabolites, 31 showed significant effects of at least one factor. GA_3_ treatment significantly affected 25 metabolites, while light intensity significantly affected 14, with 9 metabolites showing significant main effects of both factors. Significant interactions between light intensity and GA_3_ were observed for only two metabolites, cytidine and formate.

Distinct changes in primary metabolite levels were observed following GA_3_ treatment (Figure 3B). Under both light conditions, GA_3_ treatment was generally associated with increased carbohydrate levels and decreased amino acid levels. Specifically, glucose, fructose, sucrose, and xylose exhibited higher levels following GA_3_ treatment, whereas GABA, arginine, glutamine, lysine, isoleucine, valine, phenylalanine, and tyrosine exhibited lower levels.

Light intensity had more selective effects on primary metabolites. Myo-inositol and glycerate exhibited the most significant responses to light intensity. Although both metabolites were significantly affected by both factors, their F-values were substantially higher for light intensity than for GA_3_ treatment. Aspartate was the only amino acid significantly affected by light intensity but not by GA_3_ treatment, showing consistently higher levels under high-light conditions. In addition, several metabolites exhibited significant main effects of both factors without significant interaction effects. For example, glucose and xylose were strongly affected by GA_3_ treatment (adjusted *p* < 0.001) and also showed significant responses to light intensity. Both metabolites exhibited lower levels under high-light conditions while retaining the increases associated with GA_3_ treatment.

### 2.4. Effects of GA_3_ and Light Intensity on Ginsenosides

**Figure 4:**
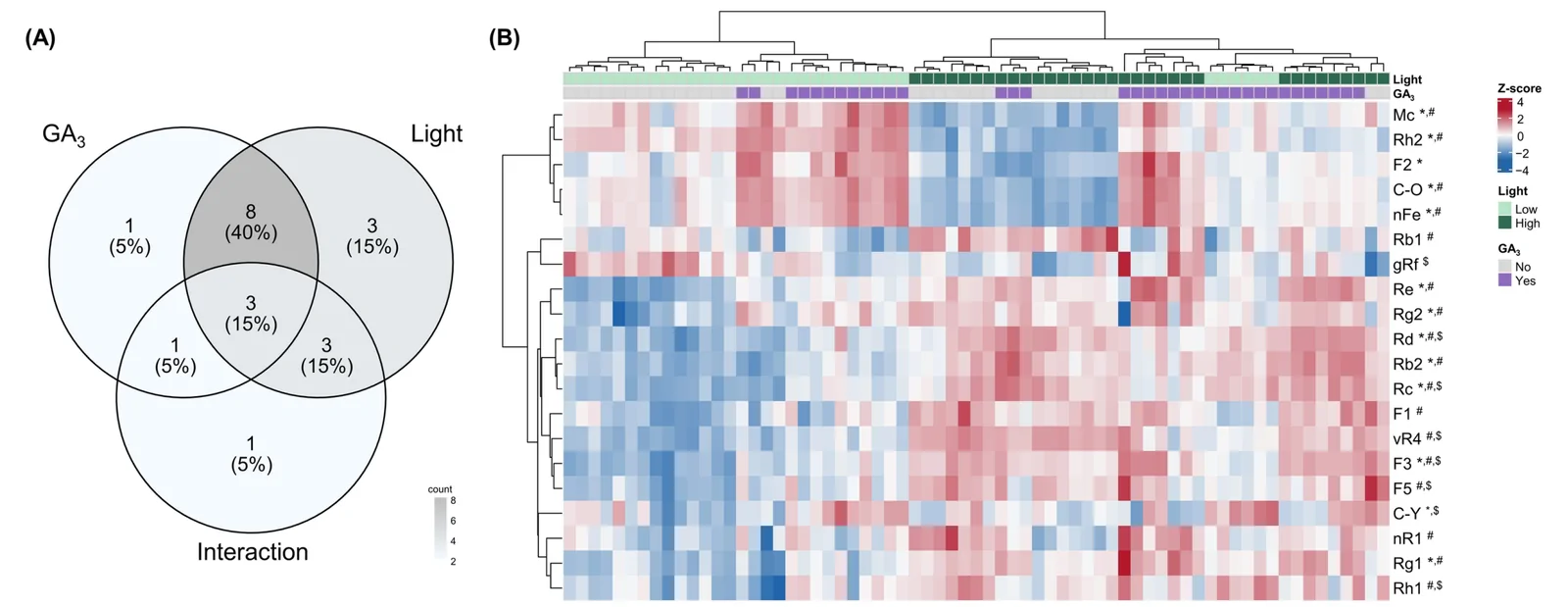
Two-way ANOVA results and heatmap of significantly affected ginsenosides identified by UPLC-QTOF/MS. (**A**) Venn diagram showing the numbers of ginsenosides significantly affected by GA_3_ treatment, light intensity, and their interaction. (**B**) Heatmap of significantly affected ginsenosides. Ginsenoside levels were scaled by row (Z-score). *, significant GA3 effect; #, significant light effect; $, significant interaction (adjusted *p* < 0.05).

Among the 26 identified ginsenosides, 20 showed significant main or interaction effects (Figure 4A and Table 2). Light intensity significantly affected 17 ginsenosides, whereas GA_3_ treatment significantly affected 13, with 11 ginsenosides showing significant main effects of both factors. For most ginsenosides significantly affected by both factors, the F-values for light intensity were substantially higher than those for GA_3_ treatment. The heatmap revealed distinct differences in ginsenoside levels between the two light conditions (Figure 4B). Several major ginsenosides, including Re, Rg1, F3, Rd, Rb2, and Rc, exhibited higher levels under high-light conditions. In contrast, Rh2, Mc, compound O (C-O), and notoginsenoside Fe (nFe) exhibited lower levels under high-light conditions. These four ginsenosides are classified as PPD-type ginsenosides lacking the Glc(1 → 2)Glc (sophorosyl) moiety at C-3, bearing either a single glucose residue (Rh2, C-O, and nFe) or no sugar moiety (Mc) at this position. Significant interaction effects between light intensity and GA_3_ were observed for eight ginsenosides, namely 20-O-glucoginsenoside Rf (gRf), compound Y (C-Y), F3, F5, Rc, Rd, Rh1, and vinaginsenoside R4 (vR4), suggesting that the responses to GA_3_ treatment varied depending on light intensity.

### 2.5. Relationships Between Primary Metabolite and Ginsenoside Levels

**Figure 5:**
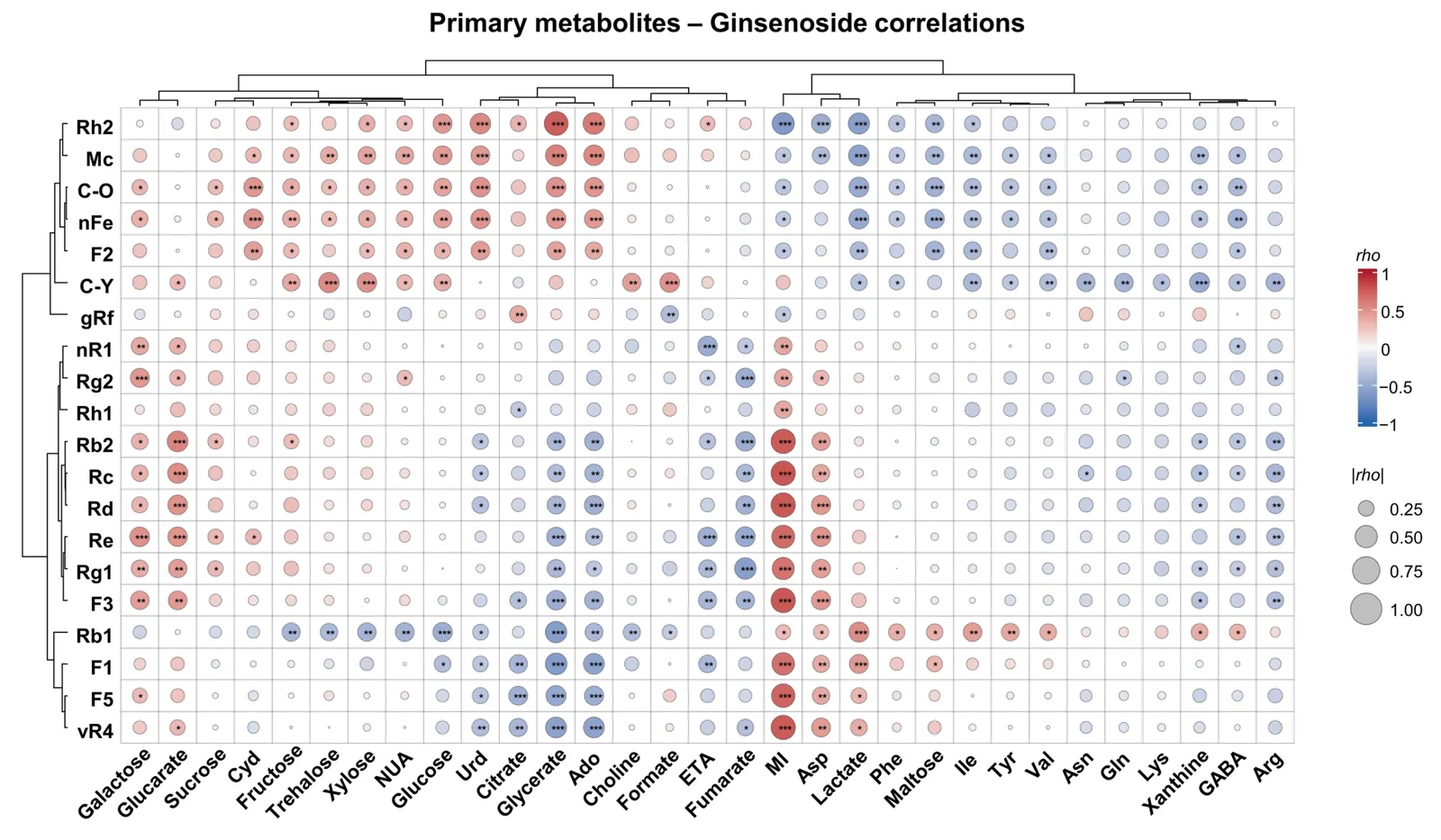
Correlation analysis between significantly affected primary metabolites and ginsenosides in ginseng sprouts. Circle color indicates the direction and magnitude of the correlation coefficient (*rho*), with red and blue representing positive and negative correlations, respectively, and circle size representing the absolute correlation coefficient (|*rho*|). *, adjusted *p* < 0.05; **, adjusted *p* < 0.01; ***, adjusted *p* < 0.001. Metabolite abbreviations are defined in Table S1 and ginsenoside abbreviations in Table 2.

To investigate the relationships between primary metabolite and ginsenoside levels across the experimental conditions, Spearman correlation analysis was performed using primary metabolites and ginsenosides showing significant responses to light intensity, GA_3_ treatment, or their interaction (Figure 5). Significant correlations were observed between several primary metabolites and ginsenosides. Strong correlations were particularly evident between light-responsive ginsenosides and primary metabolites significantly affected by light intensity, including myo-inositol, aspartate, and glycerate. In contrast, although several GA_3_-responsive primary metabolites were significantly correlated with individual ginsenosides, their overall correlation patterns were less pronounced.

**Figure 6:**
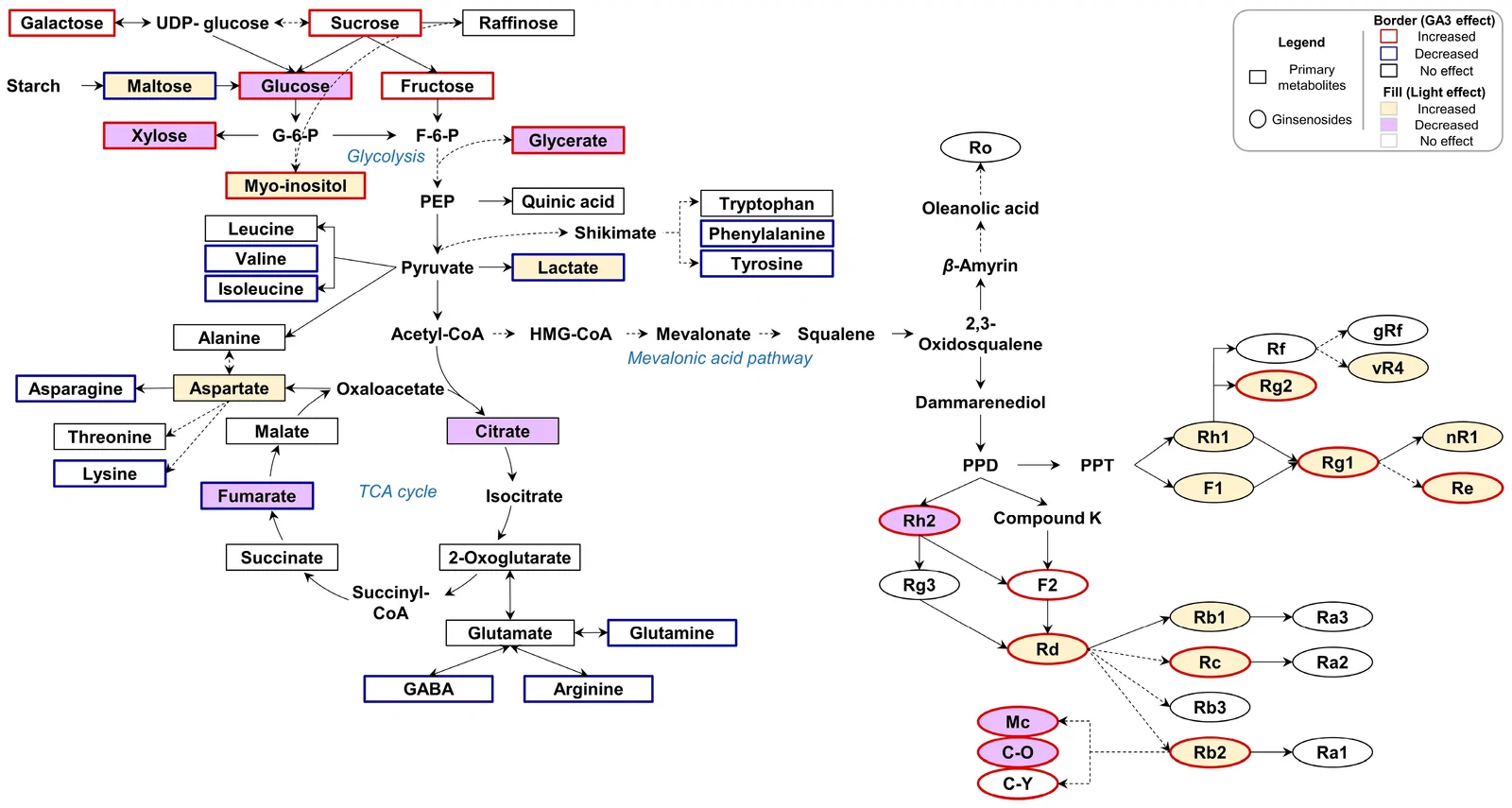
Schematic overview of primary metabolic and ginsenoside biosynthetic pathways in ginseng sprouts. Solid arrows indicate single-step conversions, whereas dashed arrows indicate multi-step or putative conversions. Rectangles represent primary metabolites and ovals represent ginsenosides. Border colors indicate the direction of the GA_3_ effect (red, increased; blue, decreased), whereas fill colors indicate the direction of the light-intensity effect (yellow, increased; purple, decreased), based on two-way ANOVA (adjusted *p* < 0.05).

To provide an integrated overview of the metabolic responses to light intensity and GA_3_ treatment, metabolites significantly affected by either factor were mapped onto primary metabolic and ginsenoside biosynthetic pathways (Figure 6). The resulting metabolic map illustrates widespread changes in primary metabolism associated with GA_3_ treatment and the pronounced differences in ginsenoside profiles associated with light intensity.

## 3. Discussion

The growth and metabolic responses observed in this study indicate that GA_3_ treatment and light intensity differentially influence ginseng sprouts. GA_3_ treatment significantly increased plant height and shoot dry weight, with a slight increase in leaf number, regardless of light intensity. These findings are consistent with the well-established role of gibberellins in promoting cell elongation and vegetative growth [11]. Similarly, GA_3_ treatment has been reported to promote shoot growth in American ginseng sprouts, with relatively limited effects on root biomass [12]. In contrast, high-light conditions were associated with reduced plant height but increased root dry weight. This pattern is consistent with previous reports linking increased light availability to dry matter accumulation and biomass allocation [20], as well as studies reporting changes in root biomass in response to the light environment in *P. ginseng* [8,9,10]. These contrasting growth responses were accompanied by distinct changes in metabolic profiles.

GA_3_ exerted a broader influence on primary metabolism, whereas light intensity had a more pronounced influence on ginsenoside profiles. Within primary metabolism, the most prominent response to GA_3_ treatment involved contrasting changes in soluble carbohydrate and free amino acid levels. The increase in soluble carbohydrate levels is consistent with previous reports suggesting that gibberellins can influence source–sink relationships and carbohydrate metabolism under growth-promoting conditions [21,22]. The decrease in free amino acid levels may reflect increased utilization during GA_3_-stimulated growth, as previously suggested in *Arabidopsis* [23]. In addition, increased soluble protein content following GA_3_ treatment has been reported in American ginseng sprouts [12]. Together, these observations suggest a possible shift in carbon and nitrogen allocation associated with GA_3_-induced shoot growth. In contrast to other soluble sugars, maltose levels decreased following GA_3_ treatment. Given that maltose is a major product of transitory starch degradation and is subsequently metabolized after export from the chloroplast [24], this decrease may reflect changes in starch-derived carbon turnover rather than an overall reduction in carbohydrate availability.

Among the primary metabolites responsive to light intensity, aspartate is involved in linking carbon and nitrogen metabolism through the transamination of oxaloacetate derived from phosphoenolpyruvate carboxylase (PEPC) activity [25,26], while glycerate is a photorespiratory intermediate that is converted to 3-phosphoglycerate for re-entry into the Calvin–Benson cycle [27,28]. These responses are consistent with previous findings that light intensity influences photosynthetic carbon fixation and associated metabolic pathways in *P. ginseng* [10], as well as broader evidence of primary metabolic adjustments during plant acclimation to environmental conditions [29,30]. Interestingly, significant interactions between light intensity and GA_3_ treatment were not observed for most primary metabolites. For example, glucose and xylose remained higher following GA_3_ treatment despite their lower levels under high-light conditions. However, the absence of significant interaction effects does not necessarily imply that GA and light signaling operate independently, as these pathways are closely interconnected at the molecular level [13]. Rather, the results suggest that any underlying interactions did not produce detectable light-dependent differences in the effects of GA_3_ treatment on most primary metabolite levels under the experimental conditions examined.

In contrast, light intensity had a more pronounced influence on ginsenoside profiles than GA_3_ treatment, with most major ginsenosides accumulating at higher levels under high-light conditions. Previous studies have reported that light conditions influence ginsenoside accumulation in *P. ginseng* [8,9,10]. More broadly, environmental factors such as light and temperature have been reported to influence the expression of genes involved in ginsenoside biosynthesis and saponin accumulation in *Panax* species [31]. In the present study, light-associated changes in individual ginsenosides appeared to be related to their C-3 glycosylation patterns. Under high-light conditions, ginsenosides lacking the C-3 Glc(1 → 2)Glc (sophorosyl) moiety (Rh2, Mc, C-O, and nFe) exhibited lower levels, whereas those containing this moiety (Rb1, Rb2, Rc, and Rd) exhibited higher levels. This pattern was not solely associated with the total number of sugar residues, as C-O and nFe contain the same number of sugar residues as Rd. In *P. ginseng*, the C-3 sophorosyl moiety can be formed through sequential glycosylation, whereby a second glucose is added to the C-3-linked glucose of Rh2 and F2 to produce Rg3 and Rd, respectively [32,33,34]. These contrasting responses may reflect increased C-3 glycosylation of PPD-type ginsenosides under high-light conditions, potentially favoring the accumulation of sophorosylated forms. However, because UDP-glycosyltransferase (UGT) activity was not measured and deglycosylation may also influence the levels of minor ginsenosides, this hypothesis requires further investigation at the gene expression and enzyme activity levels.

Significant interaction effects were observed for eight ginsenosides, indicating that their response to GA_3_ treatment depended on light intensity. Previous studies have investigated the effects of light conditions [8,9,10] and GA_3_ treatment [12] on ginseng growth and ginsenoside accumulation separately. Although the combined effects of temperature and light intensity have been evaluated in ginseng sprouts, that study focused on growth and photosynthetic characteristics without assessing metabolite profiles [35]. In contrast, the present study examined the combined effects of light intensity and GA_3_ treatment on both primary metabolites and ginsenosides. Significant interaction effects were limited to a few primary metabolites but were more frequently observed among ginsenosides. These findings suggest that changes in ginsenoside profiles under combined cultivation conditions cannot always be predicted from the effects of individual factors alone, highlighting the importance of multifactorial experimental designs for optimizing ginseng sprout cultivation.

Correlation analysis further revealed associations between primary metabolite and ginsenoside levels across the experimental conditions, with particularly strong correlations involving metabolites responsive to light intensity. Among these metabolites, myo-inositol was of particular interest, as it increased under high-light conditions and was positively correlated with most ginsenosides that also increased under high light. Myo-inositol and its derivatives are involved in cellular signaling, membrane-associated processes, and the biosynthesis of carbohydrates associated with plant stress responses and acclimation [36,37]. Furthermore, myo-inositol has been identified as a discriminating metabolite in previous studies of *P. ginseng* grown under different conditions. Higher myo-inositol levels were reported in forest-grown ginseng than in field-cultivated ginseng, potentially reflecting differences in environmental stress [38]. Positive correlations between myo-inositol and both ginsenoside Rb1 and malonyl-ginsenoside Rb1 were also observed in ginseng berries from different cultivars [39]. In addition, myo-inositol levels varied alongside those of individual ginsenosides across harvest times in wild-simulated *P. ginseng* [19]. Together, these findings suggest that myo-inositol may serve as a potential metabolic indicator of acclimation responses associated with changes in ginsenoside profiles. However, these correlations do not establish a direct regulatory or precursor relationship between myo-inositol and ginsenoside biosynthesis but rather suggest coordinated changes in their levels in response to light intensity.

In the present study, metabolic responses were analyzed in whole ginseng sprouts harvested at a single developmental stage. Given the pronounced tissue-specific distribution of primary metabolites and ginsenosides in *Panax* species [17,40], and recent evidence that aerial and root tissues exhibit distinct seasonal patterns in primary metabolites and ginsenoside levels [19], future studies examining roots, stems, and leaves separately across developmental stages could clarify whether the responses to light intensity and GA_3_ treatment are consistent across organs or reflect organ-specific metabolic adjustments. Furthermore, expanding metabolomic coverage beyond ginsenosides to include other classes of specialized metabolites would provide a more comprehensive understanding of phytochemical responses to cultivation conditions in ginseng sprouts.

## 4. Materials and Methods

### 4.1. Chemicals and Reagents

HPLC-grade acetonitrile, methanol, and water were obtained from Merck (Darmstadt, Germany). Formic acid, deuterium oxide (D_2_O), and 3-(trimethylsilyl) propionic-2,2,3,3-*d_4_* acid sodium salt (TSP-*d_4_*) were purchased from Sigma-Aldrich (St. Louis, MO, USA).

### 4.2. Plant Materials and Experimental Treatments

One-year-old seedling roots of *P. ginseng* C.A. Meyer cv. Yeonpung (Korea Seed & Variety Service database, application No. 2000-91) were purchased from a local grower in Gongju-si, Chungcheongnam-do, Republic of Korea. Ginseng seedlings were transplanted into beds filled with 50 L of commercial ginseng-specific growing medium (myeongpum-insamsangto, Shinsung Mineral Co., Ltd., Goesan, Republic of Korea) at a planting density of 20 plants per bed. All cultivation experiments were conducted in a light- and temperature-controlled growth chamber at the National Institute of Crop and Food Science, Rural Development Administration, Republic of Korea. Plants were grown under continuous illumination (24 h light/0 h dark), and the chamber temperature was maintained at 20 °C throughout the cultivation period without separate day and night temperature settings. Relative humidity was maintained at 60–70%. CO_2_ was neither supplemented nor actively controlled, and its concentration was not monitored during cultivation.

Two light intensities, 200 and 400 μmol m^−2^ s^−1^ photosynthetic photon flux density (PPFD), were provided using white LED lighting. The two PPFD levels were selected based on previously reported photosynthetic characteristics of *P. ginseng*. Light saturation has been reported at approximately 200 μmol m^−2^ s^−1^ in several Korean ginseng genotypes, whereas Yeonpung, the cultivar used in the present study, showed a higher light saturation point of approximately 400 μmol m^−2^ s^−1^ [41,42]. Accordingly, PPFD levels of 200 and 400 μmol m^−2^ s^−1^ were used as relatively lower and higher light conditions, respectively, to evaluate light-dependent differences in growth and metabolic profiles. GA_3_ was applied as a soil drench at 7-day intervals beginning on the day of transplanting. For each application, 1.6 g of IAP Gibberellin SG (3.1% gibberellic acid; Jahngryu Industries Co., Ltd., Cheongju, Republic of Korea) was freshly dissolved in 1.6 L of water, and 400 mL of this solution was diluted with 3.6 L of water to prepare 4 L of working solution per bed, corresponding to 8.95 μM GA_3_. The working solution was prepared immediately before use and was not stored. Control beds received 4 L of water per bed on the same schedule. Four beds were used, one for each combination of light intensity and GA_3_ treatment, and all beds were maintained under identical conditions except for the assigned treatments. The four experimental groups were therefore defined as follows: G1, 200 μmol m^−2^ s^−1^ without GA_3_; G2, 200 μmol m^−2^ s^−1^ with 8.95 μM GA_3_; G3, 400 μmol m^−2^ s^−1^ without GA_3_; and G4, 400 μmol m^−2^ s^−1^ with 8.95 μM GA_3_.

Individual ginseng sprout plants were used as the unit of biological replication for metabolomic analyses. The final sample sizes were G1 (*n* = 16), G2 (*n* = 18), G3 (*n* = 16), and G4 (*n* = 17). After 45 days of cultivation, ginseng sprouts were harvested, growth parameters were measured, and the samples were freeze-dried and ground into a fine powder for subsequent analyses.

### 4.3. NMR Analysis

A 100 mg aliquot of powdered whole ginseng sprout sample was suspended in 1 mL of 30% (*v*/*v*) methanol and ultrasonically extracted at 25 °C for 30 min. The extract was centrifuged at 13,000× *g* for 10 min at 4 °C, and the supernatant was filtered through a 0.22 μm syringe filter. An aliquot (600 μL) of the filtered supernatant was transferred to a glass vial and lyophilized to dryness. The dried extract was reconstituted in 500 μL of 100 mM sodium phosphate buffer prepared in D_2_O (pH 7.4) containing 2 mM TSP-*d_4_* as a chemical shift and quantification reference. The reconstituted samples were transferred to 5 mm NMR tubes.

All NMR spectra were acquired using a 700.40 MHz AVANCE III HD NMR spectrometer (Bruker BioSpin, Rheinstetten, Germany) at the KBSI Ochang Center, Republic of Korea. One-dimensional ^1^H NMR spectra were acquired at 298 K using 128 scans, a relaxation delay of 2 s, and an acquisition time of 1.704 s. Two-dimensional ^1^H–^1^H correlation spectroscopy (COSY) and ^1^H–^13^C heteronuclear single quantum coherence (HSQC) experiments were additionally performed to support metabolite identification and resolve overlapping resonances.

All ^1^H NMR spectra were manually corrected for phase and baseline. The spectra were binned over the range of 0.5–10.0 ppm using a bin width of 0.01 ppm, with the residual water region excluded from subsequent analysis. The binned spectral data were normalized to the total spectral area. Metabolite identification and quantification were performed using the Profiler module of Chenomx NMR Suite Professional (version 8.4, Chenomx Inc., Edmonton, AB, Canada) by fitting library reference spectra to the experimental spectra. Metabolite concentrations were calculated relative to the known concentration of the internal standard (TSP-*d_4_*). The metabolite concentrations were normalized by probabilistic quotient normalization (PQN).

### 4.4. UPLC-QTOF/MS Analysis

For UPLC-QTOF/MS analysis, a 100 mg aliquot of powdered sample was suspended in 1 mL of 70% (*v*/*v*) methanol and ultrasonically extracted at 25 °C for 30 min. The extract was centrifuged at 13,000× *g* for 10 min at 4 °C, and the supernatant was filtered through a 0.22 μm syringe filter.

UPLC was performed using a Waters ACQUITY I-Class UPLC system (Waters Corp., Milford, MA, USA) equipped with a Thermo Hypersil Gold column (100 mm × 2.1 mm, 1.9 μm; Thermo Fisher Scientific, Waltham, MA, USA). The column temperature was maintained at 40 °C. The mobile phase consisted of water containing 0.1% (*v*/*v*) formic acid (solvent A) and acetonitrile containing 0.1% (*v*/*v*) formic acid (solvent B). The gradient program was as follows: 0–0.5 min, 15% B; 0.5–1 min, 15–20% B; 1–6 min, 20% B; 6–13 min, 20–30% B; 13–23 min, 30–35% B; 23–24 min, 35–38% B; 24–27 min, 38–60% B; 27–31 min, 60–90% B; 31–32 min, 90–15% B; and 32–34 min, 15% B. The flow rate was 0.5 mL/min, and the injection volume was 2 μL. Mass spectrometric analysis was performed using a Xevo G2-S quadrupole time-of-flight mass spectrometer (Waters Corp., Milford, MA, USA) operated in negative electrospray ionization mode (ESI^−^). Data were acquired in MS^E^ mode with alternating low- and high-energy scans. The operating parameters were as follows: source temperature, 120 °C; desolvation temperature, 550 °C; cone gas flow, 30 L/h; desolvation gas flow, 800 L/h; capillary voltage, 2.2 kV; cone voltage, 40 V; mass range, *m*/*z* 100–2000; and collision energy, 20–45 eV. Leucine-enkephalin (*m*/*z* 554.2615 in ESI^−^ mode) was used as the lock-mass reference for accurate mass measurement.

UPLC-QTOF/MS data were processed using UNIFI (version 1.8, Waters Corp., Milford, MA, USA) and Progenesis QI (version 2.3, Waters Corp., Milford, MA, USA). Ginsenosides were identified by comparing their retention times (difference ≤ 0.4 min) and accurate masses (mass error < 5 ppm) with those of reference standards in our in-house library of 62 ginsenosides [5,43], using UNIFI 1.8. The reference standards were isolated and structurally characterized in our laboratory or obtained commercially, as described previously [5]. Progenesis QI was used for peak detection, retention-time alignment, and normalization of the metabolomic data. The processed data were subsequently exported as a data matrix containing retention time, *m*/*z*, and normalized peak abundance for statistical analysis.

### 4.5. Data Analysis

Multivariate and univariate statistical analyses were performed using the *ropls* package (version 1.40.0, Bioconductor) in R software (version 4.5.1, R Foundation for Statistical Computing, Vienna, Austria) [44] and MetaboAnalyst 6.0 (http://www.metaboanalyst.ca) [45], respectively. Prior to multivariate analysis, the data were log_10_-transformed and Pareto-scaled. PLS-DA was used to evaluate differences in overall metabolic profiles among the experimental groups. The number of components was determined automatically based on 7-fold cross-validation, and model overfitting was assessed by permutation testing with 100 permutations. Prior to univariate analyses, all data were log_10_-transformed to improve normality and homogeneity of variances, which were assessed using the Shapiro–Wilk test on model residuals and the Brown–Forsythe test, respectively. The effects of light intensity, GA_3_ treatment, and their interaction on individual metabolites were evaluated using two-way ANOVA (Type II sums of squares; F_1,63_). Spearman correlation analysis was performed to assess associations between primary metabolites and ginsenosides that were significantly affected by at least one experimental factor. Multiple-testing correction was performed using the Bonferroni method separately for each ANOVA effect within each dataset (growth parameters, primary metabolites, and ginsenosides) and across all metabolite–ginsenoside pairs for the correlation analysis. An adjusted *p* value < 0.05 was considered statistically significant. Statistical visualizations were generated using R software.

## 5. Conclusions

This study demonstrated that GA_3_ treatment and light intensity differentially influenced the growth and metabolism of ginseng sprouts, with each factor exerting distinct effects on primary metabolite and ginsenoside profiles. GA_3_ treatment primarily promoted shoot growth and had a broader influence on primary metabolism, increasing soluble carbohydrate levels and decreasing free amino acid levels under both light conditions. In contrast, high-light conditions increased root dry weight, while light intensity had a more selective effect on primary metabolites and a more pronounced influence on ginsenoside profiles. Significant interaction effects between the two factors were observed for only a few primary metabolites but were more frequent among ginsenosides, suggesting that ginsenoside responses to GA_3_ treatment were more dependent on light intensity. Correlation analysis further revealed associations between light-responsive primary metabolites, particularly myo-inositol, and ginsenoside levels across the experimental conditions. These findings highlight the importance of considering light intensity and GA_3_ treatment jointly when developing cultivation strategies to balance growth performance and ginsenoside composition in ginseng sprouts.

## Figures and Tables

**Table 1:** Effects of light intensity and GA_3_ treatment on growth parameters of ginseng sprouts. Values are presented as mean ± SD. G1, 200 μmol m^−2^ s^−1^ without GA_3_; G2, 200 μmol m^−2^ s^−1^ with GA_3_; G3, 400 μmol m^−2^ s^−1^ without GA_3_; G4, 400 μmol m^−2^ s^−1^ with GA_3_. F and adjusted *p* values (F_1,63_; Bonferroni-corrected) were obtained by two-way ANOVA.

Parameter	G1	G2	G3	G4	Light Effect		GA_3_ Effect		Interaction Effect	
					F	Adjusted *p*	F	Adjusted *p*	F	Adjusted *p*
Leaf number (no.)	13.38 ± 2.17	14.33 ± 2.00	12.88 ± 1.98	14.44 ± 1.80	0.18	1.00	6.36	0.056	0.31	1.00
Plant height (cm)	31.88 ± 2.62	35.44 ± 2.59	28.41 ± 2.72	33.72 ± 2.49	14.78	0.001	50.97	<0.001	3.47	0.27
Shoot dry weight (g)	0.29 ± 0.06	0.38 ± 0.09	0.27 ± 0.06	0.40 ± 0.07	0.04	1.00	38.24	<0.001	1.54	0.88
Root dry weight (g)	0.42 ± 0.09	0.38 ± 0.10	0.46 ± 0.09	0.47 ± 0.09	7.09	0.039	0.56	1.00	1.28	1.00

**Table 2:** Identification and relative abundance of ginsenosides in ginseng sprouts analyzed by UPLC-QTOF/MS. Values are expressed as mean peak intensities (counts) ± standard deviation. G1, 200 μmol m^−2^ s^−1^ without GA_3_; G2, 200 μmol m^−2^ s^−1^ with GA_3_; G3, 400 μmol m^−2^ s^−1^ without GA_3_; G4, 400 μmol m^−2^ s^−1^ with GA_3_. F and adjusted *p* values (F_1,63_; Bonferroni-corrected) were obtained by two-way ANOVA.

No	RT (min)	Component Name	Formula	Observed *m/z*	Mass Error (ppm)	Adducts	G1	G2	G3	G4	Light (F, adj. *p*)	GA_3_ (F, adj. *p*)	Interaction (F, adj. *p*)
1	3.74	20-O-Glucoginsenoside Rf (gRf)	C_48_H_82_O_19_	1007.5448	1.56	+HCOO, -H	16,811 ± 1985	14,181 ± 1494	12,932 ± 1810	16,076 ± 2741	4.05, 1.00	0.40, 1.00	35.41, <0.001
2	4.02	Notoginsenoside R1 (nR1)	C_47_H_80_O_18_	977.5341	1.47	+HCOO, -H	20,391 ± 1443	21,638 ± 1226	22,313 ± 2288	23,307 ± 1547	19.06, <0.001	8.41, 0.13	0.14, 1.00
3	5.59	Ginsenoside Rg1 (Rg1)	C_42_H_72_O_14_	845.4915	1.29	+HCOO, -H	670,190 ± 72,728	785,014 ± 65,543	844,722 ± 54,299	983,627 ± 128,993	92.63, <0.001	41.98, <0.001	0.07, 1.00
4	5.96	Ginsenoside Re (Re)	C_48_H_82_O_18_	991.5505	2.2	+HCOO, -H	1,183,615 ± 69,778	1,375,097 ± 44,929	1,452,494 ± 46,651	1,611,638 ± 111,481	202.69, <0.001	100.25, <0.001	3.74, 1.00
5	11.28	Vinaginsenoside R4 (vR4)	C_48_H_82_O_19_	1007.5446	1.36	+HCOO, -H	32,826 ± 2832	40,372 ± 3038	55,239 ± 2772	49,459 ± 6387	242.16, <0.001	4.32, 1.00	51.38, <0.001
6	12.17	Ginsenoside Rf (Rf)	C_42_H_72_O_14_	845.4911	0.82	+HCOO, -H	93,245 ± 9715	94,133 ± 8587	93,595 ± 12,408	92,690 ± 12,342			
7	13.44	Notoginsenoside R4 (nR4)	C_59_H_100_O_27_	1285.6491	4.43	+HCOO, -H	7654 ± 2068	7810 ± 2220	8213 ± 2770	7495 ± 3341			
8	13.52	Ginsenoside F5 (F5)	C_41_H_70_O_13_	815.4805	0.81	+HCOO, -H	157,618 ± 12,389	181,440 ± 15,014	214,086 ± 21,188	199,364 ± 24,348	71.42, <0.001	2.33, 1.00	21.92, <0.001
9	13.63	Ginsenoside Rh1 (Rh1)	C_36_H_62_O_9_	683.4378	0.32	+HCOO, -H	28,710 ± 3886	34,633 ± 4310	35,917 ± 3170	34,496 ± 3904	13.85, 0.011	6.56, 0.34	15.72, 0.005
10	13.77	Ginsenoside Rg2 (Rg2)	C_42_H_72_O_13_	829.4962	0.85	+HCOO, -H	123,318 ± 12,586	145,460 ± 10,834	150,128 ± 9006	151,678 ± 19,092	20.26, <0.001	11.61, 0.026	10.58, 0.052
11	13.96	Ginsenoside F3 (F3)	C_41_H_70_O_13_	815.4806	0.93	+HCOO, -H	188,531 ± 15,998	234,859 ± 16,198	279,595 ± 23,633	294,277 ± 26,198	231.71, <0.001	46.56, <0.001	18.02, 0.002
12	14.64	Ginsenoside Ra2 (Ra2)	C_58_H_98_O_26_	1255.6376	3.8	+HCOO, -H	29,293 ± 7832	28,124 ± 8421	30,176 ± 10,647	23,254 ± 8860			
13	14.81	Ginsenoside Ra3 (Ra3)	C_59_H_100_O_27_	1285.648	3.58	+HCOO, -H	25,364 ± 6496	22,937 ± 5546	25,617 ± 8432	20,096 ± 8230			
14	14.84	Ginsenoside Rb1 (Rb1)	C_54_H_92_O_23_	1153.6039	2.39	+HCOO, -H	179,501 ± 15,099	166,710 ± 16,725	206,179 ± 23,651	189,519 ± 20,538	26.16, <0.001	9.50, 0.078	0.030, 1.00
15	15.59	Ginsenoside Rc (Rc)	C_53_H_90_O_22_	1123.593	2.16	+HCOO, -H	318,687 ± 18,637	388,756 ± 44,348	431,099 ± 31,440	439,934 ± 49,995	80.50, <0.001	20.98, <0.001	14.55, <0.001
16	15.82	Ginsenoside Ra1 (Ra1)	C_58_H_98_O_26_	1255.6376	3.8	+HCOO, -H	40,742 ± 10,043	42,138 ± 11,845	43,871 ± 10,839	36,433 ± 12,722			
17	15.84	Ginsenoside F1 (F1)	C_36_H_62_O_9_	683.4377	0.17	+HCOO, -H	138,305 ± 18,197	150,639 ± 20,089	191,819 ± 18,769	180,730 ± 15,617	85.51, <0.001	0.25, 1.00	6.73, 0.31
18	15.85	Ginsenoside Ro (Ro)	C_48_H_76_O_19_	955.4922	1.46	+HCOO, -H	28,074 ± 5239	22,101 ± 5506	27,747 ± 5413	27,800 ± 4337			
19	16.54	Ginsenoside Rb2 (Rb2)	C_53_H_90_O_22_	1123.5931	2.25	+HCOO, -H	370,907 ± 27,066	435,048 ± 61,130	48,3974 ± 31,378	536,460 ± 66,164	79.04, <0.001	21.86, <0.001	1.05, 1.00
20	18.54	Ginsenoside Rd (Rd)	C_48_H_82_O_18_	991.5504	2.1	+HCOO, -H	1,035,639 ± 54,584	1,181,359 ± 91,524	1,280,687 ± 66,461	1,310,642 ± 97,126	93.23, <0.001	22.56, <0.001	11.27, 0.026
21	22.15	Notoginsenoside Fe (nFe)	C_47_H_80_O_17_	961.5389	1.19	+HCOO, -H	161,231 ± 27,585	185,819 ± 35,682	109,529 ± 18,123	175,436 ± 44,538	20.50, <0.001	36.06, <0.001	10.42, 0.052
22	23.46	Compound O (C-O)	C_47_H_80_O_17_	961.5385	0.78	+HCOO, -H	197,986 ± 34,692	229,098 ± 44,346	136,148 ± 20,902	211,199 ± 55,276	21.36, <0.001	31.98, <0.001	7.89, 0.18
23	25.37	Ginsenoside F2 (F2)	C_42_H_72_O_13_	829.4968	1.58	+HCOO, -H	288,386 ± 29,535	328,275 ± 48,682	253,464 ± 23,902	306,845 ± 63,310	8.10, 0.15	17.37, <0.001	0.51, 1.00
24	26.5	Ginsenoside Mc (Mc)	C_41_H_70_O_12_	799.4853	0.46	+HCOO, -H	3047 ± 398	3394 ± 476	2283 ± 384	2888 ± 505	39.79, <0.001	22.14, <0.001	3.10, 1.00
25	26.66	Compound Y (C-Y)	C_41_H_70_O_12_	799.4853	0.46	+HCOO, -H	2726 ± 303	3242 ± 275	2925 ± 278	2889 ± 304	1.24, 1.00	11.66, 0.026	15.36, 0.006
26	27.79	Ginsenoside Rh2 (Rh2)	C_36_H_62_O_8_	667.4423	−0.55	+HCOO	2351 ± 295	2579 ± 460	1296 ± 235	1748 ± 399	129.20, <0.001	18.24, 0.002	5.62, 0.54

## Data Availability

The original contributions presented in this study are included in the article/Supplementary Materials. Data are available from the corresponding author upon reasonable request.

## Supplementary Materials

The following supporting information can be downloaded at: https://www.mdpi.com/article/10.3390/ijms27219614/s1.

## Abbreviations

The following abbreviations are used in this manuscript:

ANOVA	Analysis of variance
BPI	Base peak intensity
ESI	Electrospray ionization
GA_3_	Gibberellic acid
GABA	4-Aminobutyrate
LED	Light-emitting diode
NMR	Nuclear magnetic resonance
PEPC	Phosphoenolpyruvate carboxylase
PLS-DA	Partial least squares discriminant analysis
PPD	Protopanaxadiol
PPFD	Photosynthetic photon flux density
PPT	Protopanaxatriol
PQN	Probabilistic quotient normalization
SD	Standard deviation
TCA	Tricarboxylic acid
TSP-*d_4_*	3-(trimethylsilyl) propionic-2,2,3,3-*d_4_* acid sodium salt
UGT	UDP-glycosyltransferase
UPLC-QTOF/MS	Ultra-performance liquid chromatography–quadrupole time-of-flight mass spectrometry
